# Enhancement of hepatocyte differentiation from human embryonic stem cells by Chinese medicine Fuzhenghuayu

**DOI:** 10.1038/srep18841

**Published:** 2016-01-06

**Authors:** Jiamei Chen, Wei Gao, Ping Zhou, Xiaocui Ma, Benjamin Tschudy-Seney, Chenghai Liu, Mark A Zern, Ping Liu, Yuyou Duan

**Affiliations:** 1 Shuguang Hospital affiliated to Shanghai University of Traditional Chinese Medicine, Institute of Liver Diseases, Shanghai University of Traditional Chinese Medicine, Shanghai, China; 2 Department of Internal Medicine, University of California Davis Medical Center, Sacramento, California, USA; 3 Institute for Regenerative Cures, University of California Davis Medical Center, Sacramento, California, USA; 4 Department of Dermatology, University of California Davis Medical Center, Sacramento, California, USA; 5 Institute of Biophysics, the Chinese Academy of Science, Beijing, China; 6 E-institutes of Shanghai Municipal Education Commission, Shanghai University of Traditional Chinese Medicine, Shanghai, China

## Abstract

Chinese medicine, Fuzhenghuayu (FZHY), appears to prevent fibrosis progression and improve liver function in humans. Here we found that FZHY enhanced hepatocyte differentiation from human embryonic stem cells (hESC). After treatment with FZHY, albumin expression was consistently increased during differentiation and maturation process, and expression of metabolizing enzymes and transporter were also increased. Importantly, expression of mesenchymal cell and cholangiocyte marker was significantly reduced by treatment with FZHY, indicating that one possible mechanism of FZHY’s role is to inhibit the formation of mesenchymal cells and cholangiocytes. Edu-labelled flow cytometric analysis showed that the percentage of the Edu positive cells was increased in the treated cells. These results indicate that the enhanced proliferation involved hepatocytes rather than another cell type. Our investigations further revealed that these enhancements by FZHY are mediated through activation of canonical Wnt and ERK pathways and inhibition of Notch pathway. Thus, FZHY not only promoted hepatocyte differentiation and maturation, but also enhanced hepatocyte proliferation. These results demonstrate that FZHY appears to represent an excellent therapeutic agent for the treatment of liver fibrosis, and that FZHY treatment can enhance our efforts to generate mature hepatocytes with proliferative capacity for cell-based therapeutics and for pharmacological and toxicological studies.

Liver disease is a major health problem in the world, and can be genetic or caused by a variety of factors that damage the liver, such as hepatitis viruses or alcohol consumption. Over time, such damage to the liver can result in fibrosis and cirrhosis^1^, a sign of liver damage and a potential contributor to liver failure through progressive cirrhosis of the liver^2^. Traditional Chinese medicines are currently used to treat patients with moderate to advanced fibrosis which were caused by chronic viral hepatitis B and C^3^,^4^, including Fuzhenghuayu (FZHY)^5^,^6^,^7^,^8^. The FZHY recipe is an SFDA-approved anti-fibrotic medicine in China^9^, and consists of six Chinese medicine herbs, namely Semen Persicae, Radix Salvia Miltiorrhizae, Gynostemma Pentaphyllammak, Cordyceps, Pollen Pini, and Fructus Schisandrae Chinensis^10^ (Suppl. Fig. 1, and Suppl. Table 1). Clinical trials in China showed that FZHY could significantly improve clinical symptoms and liver function, reverse hepatic fibrosis and decrease portal pressure in patients with chronic hepatitis B, with liver fibrosis and cirrhosis^10^,^11^,^12^,^13^. This antifibrotic effect was also demonstrated in the completion of an FDA-approved phase II clinical trial in patients with hepatitis C in the US in 2013^14^. These results indicated that FZHY can play an important role in improving liver disease, including hepatocyte function. Mimicking liver development, we have developed an efficient protocol to generate metabolically functioning hepatocytes from human embryonic stem cells (hESC)^15^ and human induced pluripotent stem cells^16^, and these hepatocytes exhibit *in vivo* function shown by engrafting and proliferation in mouse livers^16^. Our results are encouraging, however, the differentiated cells were not completed mature hepatocytes. Because of its effect in clinical conditions, we speculated that FZHY treatment might also enhance the process of hepatocyte differentiation from hESC. Our results suggest that it did.

## Results

### Enhancement of hepatocyte differentiation and maturation by FZHY

**Figure 1:**
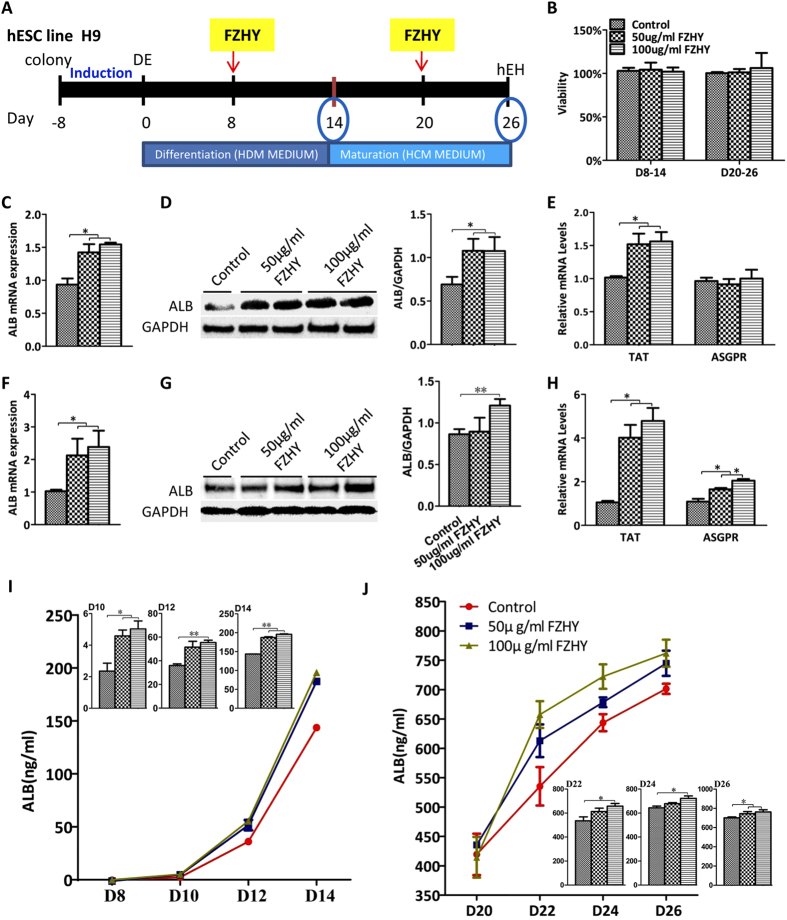
Hepatocyte differentiation of hESC under treatment with FZHY. (**A**) Schematic illustration of differentiation protocol with the addition of FZHY. Briefly, Hepatocyte differentiation was initiated after the induction of definitive endoderm (DE) using hepatocyte differentiation medium (HDM), and HDM was changed to hepatocyte culture medium (HCM) for further differentiation and maturation at day 14 after differentiation. FZHY was added into the mediums at final concentrations of 50 μg/ml and 100 μg/ml at days 8 and 20 after differentiation for six days-treatment respectively. hESC-derived hepatocytes (hEH) were harvested at days 14 and 26 after treatment with FZHY for analysis. (**B**) Cell viability was assessed by MTT assay at day 14 and day 26 respectively. (**C,F**) qPCR was employed to determine relative albumin (ALB) expression at days 14 (**C**) and 26 (**F**) of differentiation in the treated cells compared to the untreated cells. (**D,G**) ALB protein expression was determined by Western blot in the untreated and treated cells at days 14 (**D**) and 26 (**G**) (Left panel). ALB protein expression was measured employing histogram normalized to GAPDH after calculation based on the results of Western blot in the untreated and treated cells at days 14 (**D**) and 26 (**G**) (Right panel). (**E,H**) Relative expression of TAT and ASGPR was determined by qPCR at days 14 (**E**) and 26 (**H**) of differentiation in treated cells compared to untreated cells. (**I**,J) The amount of secreted ALB in the culture medium was measured by ELISA analysis at days of 14 (**I**) and 26 (**J**) of differentiation in treated and untreated cells, and the secretion of ALB in treated cells exhibited significantly higher than those in untreated cells, which were shown by the panels of column graphs. Data represent mean ± SEM. *p < 0.05, **p < 0.01.

Hepatocyte differentiation was performed as previously described^15^. In our screening tests with different concentrations of FZHY and the addition of FZHY at different time points during the differentiation process, we found that hESC-derived hepatocyte differentiation and maturation could be promoted at the concentration of 50 and 100 μg/ml FZHY and the addition times at days 8 and 20 for 6 days (Suppl. Fig. 2); thus, these parameters were employed to modify our differentiation protocol in this study (Fig. 1A). The differentiating cells were treated with FZHY between days 8–14, whereas FZHY was added between days 20–26 during the maturation process (Fig. 1A). MTT results showed that the viability of the cells treated with 50 and 100 μg/ml FZHY was not affected when compared to cells without treatment (Fig. 1B). The differentiation process was enhanced with FZHY, as determined by the increase of albumin expression. Results of qPCR showed that albumin expression in treated cells was increased when compared to the cells without treatment (Fig. 1C), and the increase of albumin was further confirmed by Western blot (Fig. 1D). The functional enzyme, tyrosine aminotransferase (TAT), was also more highly expressed in the treated cells, as determined by qPCR (Fig. 1E). In the functional assay, ELISA analysis showed that secreted albumin in the medium was increased during the period of the treatment (Fig. 1I). Albumin expression was also increased in treated cells during the maturation process (Fig. 1F,G). Expression of both TAT and asialoglycoprotein receptor (ASGPR), an important marker of mature and functional hepatocytes was also increased in treated cells when compared to control during the maturation process (Fig. 1H). Finally, ELISA analysis revealed that the secreted albumin in the medium was also increased in the treated cells even 3 weeks after differentiation (Fig. 1J), indicating that the treated cells were more mature. Taken together, these results strongly suggested that treatment with FZHY could promote and enhance the differentiation and maturation of hepatocytes derived from hESC.

### Increased expression of metabolizing enzymes and transporter

**Figure 2:**
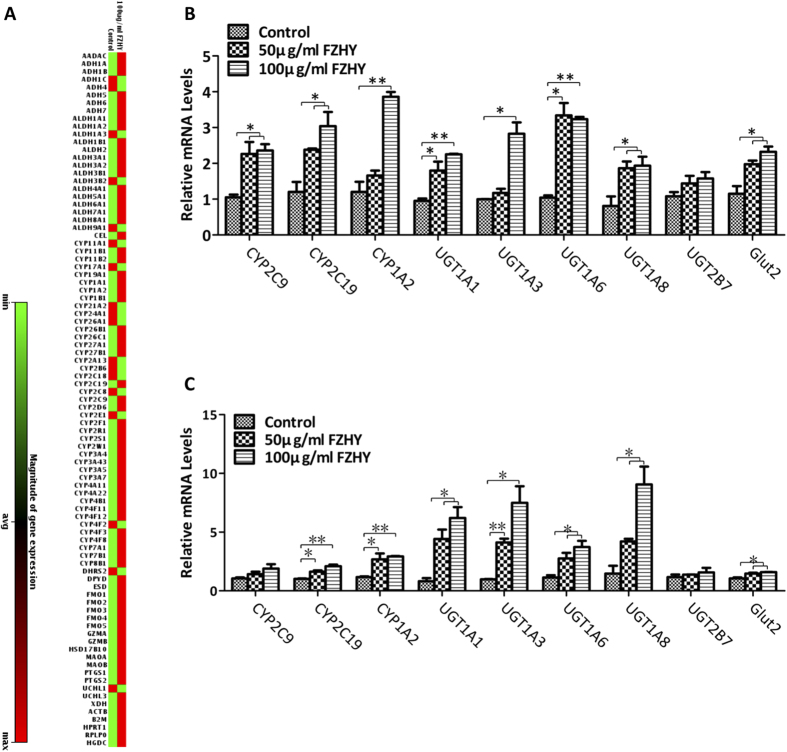
Increased expression of metabolizing enzymes and transporter. (**A**) The entire histogram view of the expression changes of 84 genes representing phase I enzymes by PCR Array analysis at day 14 after differentiation in the presence or absence of FZHY. The expression fold changes are listed in Suppl. Fig. 2. (**B,C**) Relative expression levels of phase I, II and III enzymes and proteins were determined at days 14 (**B**) and 26 (**C**) of differentiation in the treated cells and compared to untreated cells. Data represent mean ± SEM. *p < 0.05, **p < 0.01.

Metabolic function is one of the most important actions of hepatocytes. After treatment with FZHY, PCR Array analysis showed that expression of most genes from 84 phase I enzymes was increased, particularly in the group treated with 100 μg/ml FZHY when compared to those in the cells without treatment (Fig. 2A, the details of the fold change of these genes are listed in Suppl. Fig. 3). We quantitatively evaluated CYP2C9, CYP2C19, and CYP1A2 from Phase I; UGT1A1, UGT1A3, UGT1A6, UGT1A8, and UGT2B7 from phase II; and glucose transporter protein 2 (Glut 2) from phase III. qPCR results revealed that these genes were highly expressed during both differentiation and maturation processes in the treated cells when compared to cells without treatment (Fig. 2B,C). Moreover, expression levels of UGT1A1, UGT1A3, and UGT1A8 were higher during the maturation stage in the treated cells. These results demonstrated that these enzyme-mediated metabolizing function would be improved in the treated cells; thus, after treatment with FZHY, the metabolic function of hESC-derived hepatocytes was significantly increased, indicating that they were more mature.

### Enhancement of hepatocyte proliferation

**Figure 3:**
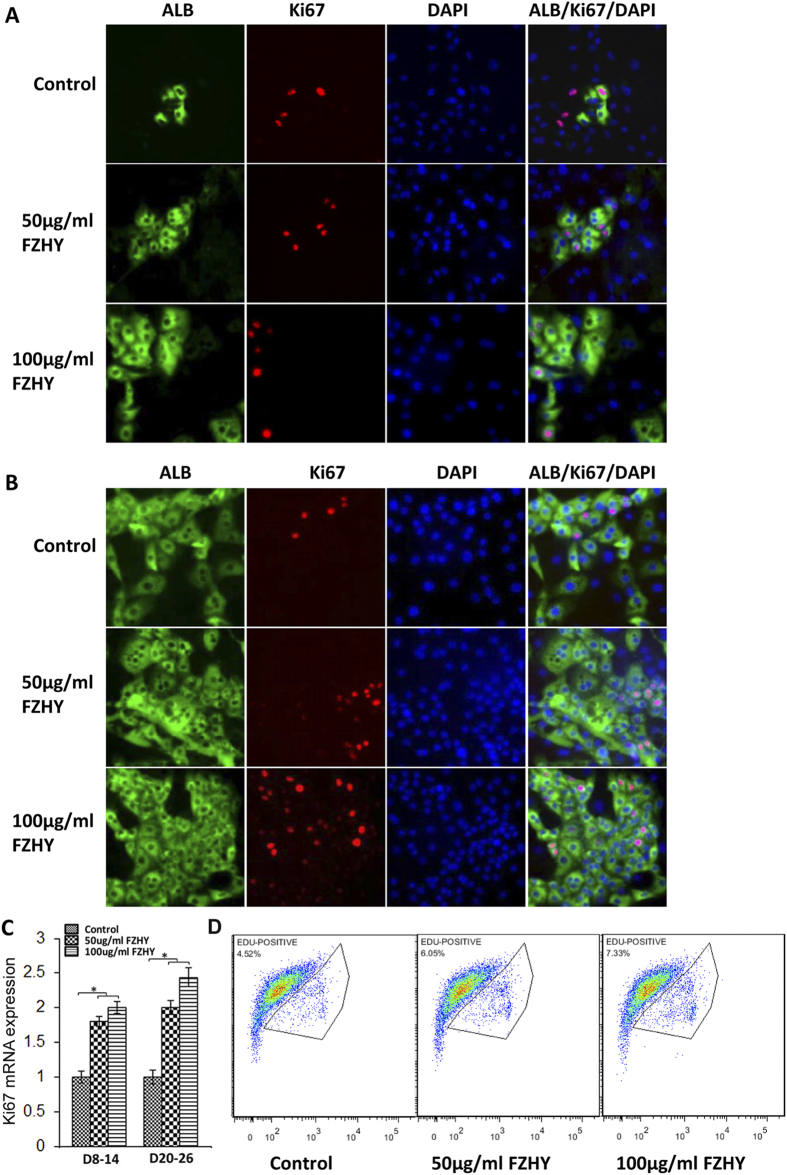
Enhancement of hepatocyte proliferation. (**A,B**) Double-immunostaining was performed to detect co-expression of albumin (ALB) and Ki67 at days 14 (**A**) and 26 (**B**) in the untreated and the treated cells. (**C**) The relative expression of Ki67 gene was determined by qPCR at days 14 and 26 in the treated cells and compared to the untreated cells. (**D**) Edu flow cytometry showed the cell number of the cells with proliferative capacity at day 26 of differentiation in treated and untreated (control) cells. Magnification: 100 x. *p < 0.05, **p < 0.01.

The lack proliferative capacity hinders the use of hepatocytes in *in vitro* culture and analysis. Our hESC-derived hepatocytes maintained proliferative ability up to day 30 after differentiation by showing co-expression of albumin and Ki67, a proliferative marker, and they maintained good growth in secondary culture (data not show). During treatment with FZHY, the proliferative capacity of hepatocytes was enhanced in the treated cells as indicated by increasing numbers of cells that were double positive for albumin and Ki67 during the differentiation and maturation processes, as determined by immunochemistry analysis (Fig. 3A,B). Results of qPCR showed that expression of Ki67 in treated cells was increased when compared to the cells without treatment during the differentiation and maturation processes (Fig. 3C). At day 26 of differentiation, Edu-labelled flow cytometric analysis results showed 4.52% of cells with proliferative capacity in controls, and the percentage of the cells with this capacity was increased to 6.05% and 7.33% in the cells treated with 50 μg/ml and 100 μg/ml of FZHY respectively (Fig. 3D). Thus, these results demonstrated that FZHY could promote hepatocyte proliferation *in vitro*.

### FZHY inhibited the formation of mesenchymal cells and cholangiocytes

**Figure 4:**
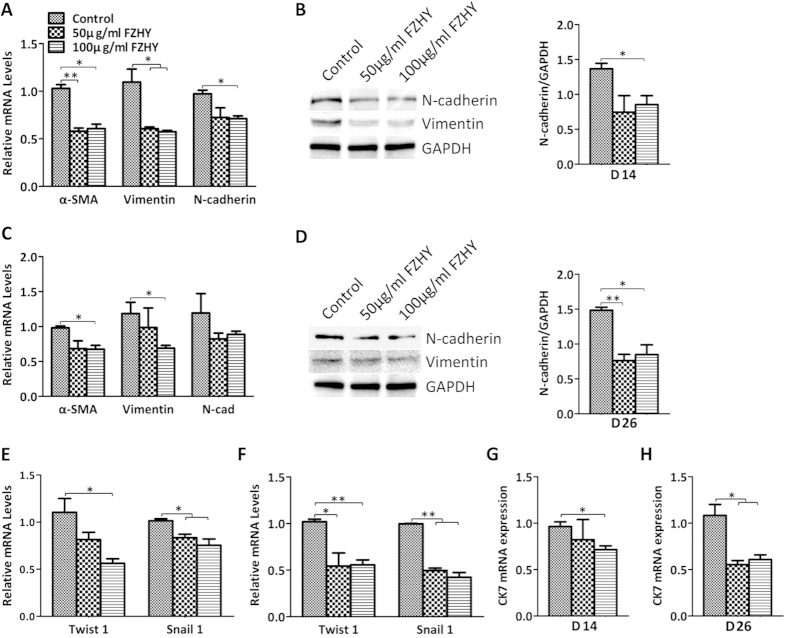
The inhibition of mesenchymal cell and cholangiocyte by treatment with FZHY. (**A,C**) The relative expression of the mesenchymal cell markers was determined by qPCR at days 14 (**A**) and 26 (**C**) in the treated cells and compared to the untreated cells. (**B,D**) Expression of N-cadherin and Vimentin was determined by Western blot at days 14 (**B**) and 26 (**D**) in the untreated and treated cells (Left panel), and the amount of N-cadherin was also measured employing histogram normalized to GAPDH protein based on the results of Western blot (Right panel). (**E,F**) The relative expression of the associated transcription factors was measured by qPCR at days 14 (**E**) and 26 (**F**) in the treated cells and compared to the untreated cells. (**G,H**) The relative expression of cholangiocyte marker was determined by qPCR at days 14 (**G**) and 26 (**H**) in the treated cells and compared to the untreated cells. *p < 0.05, **p < 0.01.

Because it had been reported previously that FZHY inhibits fibrogenesis^10^,^11^,^12^,^13^, and it appears that epithelial-to-mesenchymal transition (EMT) contributes significantly to the process of liver fibrosis^17^, we decided to evaluate the effect of FZHY on the formation of mesenchymal cells through evaluation of EMT. qPCR results showed that N-cadherin, α-SMA, and Vimentin, markers of mesenchymal cells or EMT, were reduced in the FZHY-treated cells during both the differentiation and maturation stages (Fig. 4A,C). Western blot further confirmed the reduction of N-cadherin and Vimentin (Fig. 4B,D). Moreover, EMT-associated transcription factors, Snail1, and Twist family member, Twist1, were significantly down-regulated by the treatment with FZHY (Fig. 4E,F), suggesting that FZHY inhibited the formation of mesenchymal cells through the reduction of EMT. In addition, qPCR results showed that expression of CK7, a cholangiocyte marker, was significantly decreased in the treated cells when compared to the cells without treatment during both the differentiation and maturation processes (Fig. 4G,H). These data indicated that FZHY can inhibit the formation of mesenchymal cells and cholangiocytes in the processes of differentiation and maturation of hepatocytes derived from hESC.

### Signaling pathways affected by treatment with FZHY

**Figure 5:**
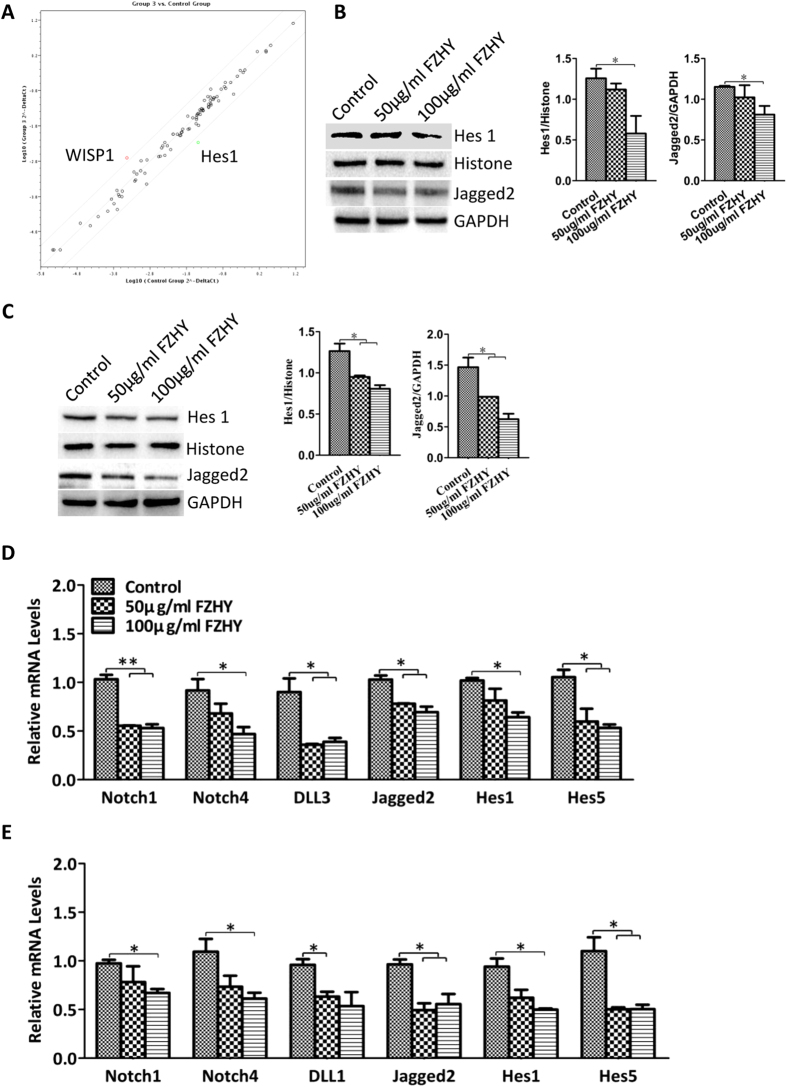
Signaling pathways affected by the treatment with FZHY. (**A**) PCR Array revealed up-regulation of Wnt signaling pathway and down-regulation of Notch signaling pathway (The details of expression of 84 genes representing 10 signaling pathways was listed in Suppl. Fig. 3). (**B,C**) Expression of Hes1 and Jagged2 were detected by Western blot at days 14 (**B**) and 26 (**C**) after differentiation in the treated cells and compared to the untreated cells, and Histone and GAPDH as housekeeping genes respectively (Left panel). The amount of Hes1 and Jagged2 was measured employing histogram normalized to Histone and GAPDH protein respectively based on the results of Western blot (Right panel). (**D,E**) Relative expression of genes from Notch pathway was determined by qPCR at days 14 (**D**) and 26 (**E**) after differentiation in the treated cells and compared to the untreated cells. Data represent mean ± SEM. *p < 0.05, **p < 0.01.

Employing the Signal Transduction PathwayFinder PCR Array kit, we found that Wnt1-inducible-signaling pathway protein 1(WISP1) was up-regulated, and Hes1, the transcription factor of Notch signaling pathway was down-regulated (Fig. 5A, the details of the fold change of 84 genes are listed in Suppl. Fig. 4). Normally the Notch pathway regulates cholangiocyte differentiation^18^. We further determined that the expressions of Notch receptor, Notch1 and Notch4, Notch ligand, DLL3 and Jagged2, and Notch target genes, Hes1 and Hes5, were down-regulated in treated cells during the differentiation process (Fig. 5D), and Notch1, Notch4, DLL1, Jagged2, Hes1 and Hes5 were down-regulated in treated cells during maturation when compared to cells without treatment (Fig. 5F). Western blot results further confirmed that Hes1 and Jagged2 were down-regulated in treated cells during both differentiation and maturation processes (Fig. 5B,C).

### FZHY enhanced both the canonical Wnt and ERK signaling pathways

**Figure 6:**
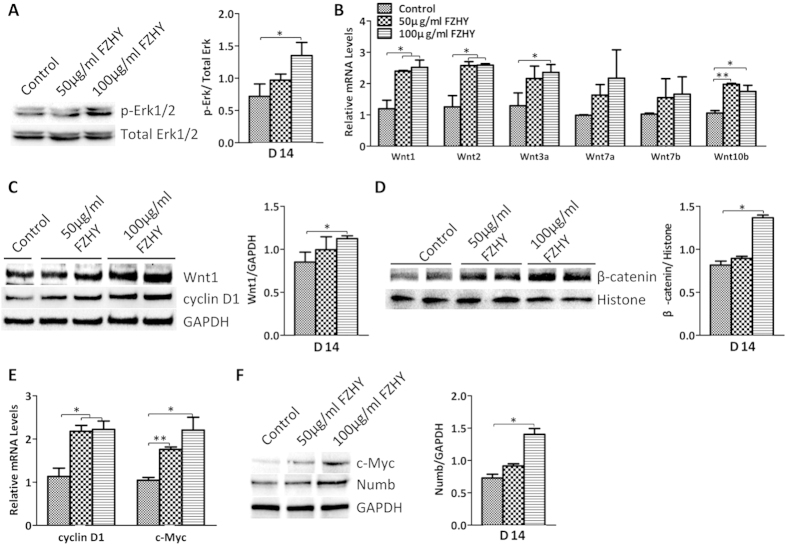
Enhancement of ERK and Wnt pathways by FZHY during differentiation. (**A**) Expression of phosphorylated Erk1/2 (p-Erk1/2) and total Erk1/2 was determined by Western blot at day 14 of differentiation in the treated and untreated cells (Left panel), and expression of p-Erk1/2 also were measured employing histogram normalized to total Erk1/2 after calculation based on the results of Western blot (Right panel). (**B**) Relative expression of genes from Wnt ligands were determined by qPCR at day 14 of differentiation in the treated and untreated cells. (**C**) Expression of Wnt1 and cyclin D1 was detected by Western blot at day 14 of differentiation in the treated and untreated cells (Left panel), and Wnt1 expression also was measured employing histogram normalized to GAPDH after calculation based on the results of Western blot (Right panel). (**D**) The amount of nuclear beta-catenin was determined by Western blot at day 14 of differentiation in the treated and untreated cells (Left panel), and its amount also was measured employing histogram normalized to Histone protein after calculation based on the results of Western blot (Right panel). (**E**) Relative expressions of cyclin D1 and c-Myc were determined by qPCR in the treated cells and compared to the untreated cells at day 14 of differentiation. (**F**) Expressions of c-Myc and Numb were determined by Western blot at day 14 of differentiation (Left panel), and the expression of Numb was also measured employing histogram normalized to GAPDH based on the results of Western blot (Right panel). Data represent mean ± SEM. *p < 0.05, **p < 0.01.

**Figure 7:**
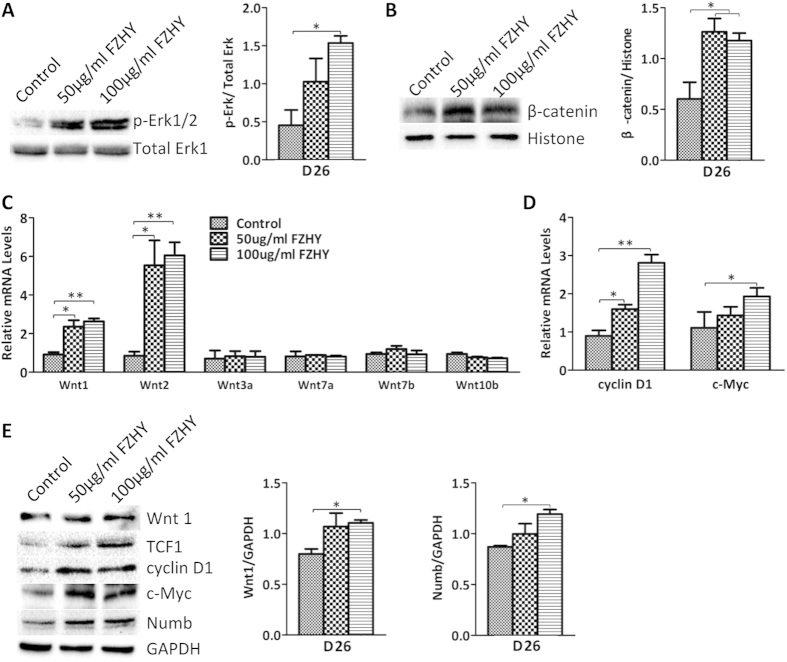
Enhancement of ERK and Wnt pathways by FZHY during maturation. (**A**) Expression of phosphorylated Erk1/2 and total Erk1/2 was determined by Western blot at day 26 after differentiation in the treated and untreated cells (Left panel), and expression of p-Erk1/2 also was measured employing histogram normalized to total Erk1/1 after calculation based on the results of Western blot (Right panel). (**B**) The amount of nuclear beta-catenin was determined by Western blot at day 26 of differentiation in the treated and untreated cells (Left panel), and its amount also was measured employing histogram normalized to Histone H3 protein after calculation based on the results of Western blot (Right panel). (**C**) Relative expression of genes from Wnt ligands were determined by qPCR at day 26 of differentiation in the treated and untreated cells. (**D**) Relative expression of cyclin D1 and c-Myc was determined by qPCR in the treated cells and compared to those in the untreated cells at day 26 of differentiation. (**E**) Expression of Wnt1, TCF1, cyclin D1, c-Myc, and Numb was determined by Western blot at day 26 of differentiation in the treated and untreated cells (Left panel), and the expression of Wnt1 and Numb also was measured employing histogram normalized to GAPDH after calculation based on the results of Western blot (Right panel). Data represent mean ± SEM. *p < 0.05, **p < 0.01.

The MAPK signaling pathway has been shown to be involved in early liver development and hepatic differentiation^18^,^19^, and ethanol treatment inhibited both the ERK/MAPK and canonical (β-catenin dependent) Wnt signaling pathways in hESC-derived hepatocytes in our previous study^20^, indicating that both signaling pathways regulate hepatocyte differentiation. Western Blot results showed that phosphorylated ERK was highly expressed in FZHY-treated cells during both the differentiation and maturation processes, whereas total ERK was not changed in the treated and untreated cells (Figs 6A and 7A). qPCR results showed that Wnt1, Wnt2, Wnt3a, and Wnt10b were more highly expressed in treated cells in the differentiation process, and expression of Wnt1 and Wnt2 was increased during the maturation process in treated cells (Figs 6B and 7C), and Western Blot results showed that Wnt1 was highly expressed in FZHY-treated cells during both the differentiation and maturation processes (Figs 6C and 7E), suggesting that FZHY treatment enhanced both ERK and Wnt signaling pathways during the whole process of hepatocyte differentiation. β-catenin nuclear translocation, and TCF1 up-regulation have been shown to be involved in both ERK and canonical Wnt pathways^21^,^22^,^23^, and in our studies, Western blot results showed that the amount of β-catenin protein in nuclei was increased in treated cells (Figs 6D and 7B), and expression of TCF1, a β-catenin target gene, was also increased (Fig. 7E). We further evaluated TCF1 downstream targets such as cyclin D1 and c-myc, which facilitate cell proliferation. Both qPCR results and Western blots revealed that both cyclin D1 and c-myc were more highly expressed in the treated cells when compared to untreated cells during both the differentiation and maturation processes (Figs 6C,E,F and 7D,E). Numb mediates the interaction between Wnt and Notch as an interruption of the Wnt-Notch signaling cycle^24^,^25^. Western blot showed that the expression of Numb was up-regulated during both differentiation and maturation processes (Figs 6F and 7E), indicating that its up-regulation is consistent with the activation of canonical Wnt signaling and suppression of Notch signaling.

## Discussion

The liver has the unique capacity to regulate its growth and mass, and this growth process, following acute and chronic liver injury, is known as liver regeneration^26^. In addition to hepatocytes and non-parenchymal cells, the liver contains intra-hepatic adult stem cells that can differentiate into hepatocytes and cholangiocytes under pathophysiological conditions. Liver regeneration after mild degrees of injury or partial hepatectomy does not involve intra- or extra-hepatic stem cells but depends on the proliferation of hepatocytes^26^. However, sustained injury will cause hepatocyte senescence, and when mature hepatocyte proliferation is exhausted or suppressed^27^,^28^, this condition results in activation of hepatic progenitor cells. When the need for proliferation of hepatocytes is overwhelmed, the loss of mature hepatocytes and restoration of the liver mass and function appears to be compensated for by involvement of liver progenitor or stem cells that proliferate and differentiate into ductular cells or hepatocytes^26^,^29^. Intrinsic and extrinsic factors favoring differentiation of liver progenitor/stem cells will promote the restoration of liver function.

In liver development, fibroblast growth factor (FGF) from the cardiac mesoderm and bone morphogenetic proteins (BMPs) from septum transversum mesenchymal cells coordinately induce the liver differentiation and suppress the pancreatic differentiation^19^,^30^,^31^,^32^,^33^ in the ventral foregut. MAPK is activated in response to FGF in the lateral hepatic progenitors^18^,^19^. Recent reports indicate an important role of FGF/ERK signaling in promoting the transition from a naϊve state to a primed state in pluripotent stem cells^30^,^34^,^35^,^36^,^37^,^38^. FGF4 is the major stimulus activating ERK in embryonic stem cells (ESC), and FGF4 stimulation of ERK1/2 is an autoinductive stimulus to induce ESC to differentiate, thus, it appears that the ERK cascade directs the transition to a state that is responsive to inductive cues for germ layer segregation^39^, and further investigation has shown that FGF4 signaling controls endoderm lineages^34^. In addition, a recent study showed that FGF4 and HGF promote the differentiation of mouse bone marrow mesenchymal stem cells towards hepatocytes via the MAPK (p-38 and ERK) pathway^40^.

In our differentiation protocol, FGF4, HGF, BMP2 and BMP4 were used to induce early hepatic differentiation^15^,^16^, mimicking *in vivo* liver development; thus, it is expected that MAPK is one of the major signaling pathways to regulate hepatic differentiation under our conditions. Our recent study showed ethanol negatively regulated hepatic differentiation from hESC by the inhibition of the MAPK/ERK signaling pathway^20^; these results suggest that the activation of ERK is a critical pathway which directs the hepatic progenitors to differentiate towards hepatocytes under our differentiation condition.

The Wnt signaling pathway appears to promote hepatogenesis in multiple systems including xenopus^41^, and Zebrafish^42^,^43^,^44^. In mammalian liver development, mesenchymal origin Wnt and FGF4 signaling in the foregut enables liver and pancreas induction^19^,^41^,^45^. Investigations in rodents elucidated the role of Wnt1 in directing oval cells to differentiate to hepatocytes during liver regeneration after liver injury in rats^46^. This suggests that the Wnt signaling pathway also plays an important role in hepatocyte differentiation from stem cells. Our latest study revealed that ethanol impaired hepatocyte differentiation from hESC by reducing the canonical Wnt signaling pathway^20^. Thus the results from both studies suggest that Wnt signaling pathway also is involved in regulating hepatocyte differentiation from hESC under our differentiation conditions.

FZHY appears to prevent the progression of fibrosis and to improve liver function in humans with liver disease^10^,^11^,^12^. In this study, we determined that FZHY could promote and enhance both hepatocyte differentiation and maturation under our differentiation conditions, including the secretion of albumin into the media (Fig. 1). This result was consistent with the finding of a clinical trial; when patients with liver fibrosis caused by chronic hepatitis B were treated with FZHY, FZHY improved liver function parameters, particularly an increase of albumin levels^47^. Therefore, animal experiments are expected to be performed in the further investigations using a liver injury model to determine whether treatment with FZHY also plays similar roles after transplantation of hESC-derived hepatocytes into injured livers.

Our results also showed that FZHY could promote hepatocyte proliferation, as indicated by the increased number of cells that co-expressed albumin and Ki67 and the increased percentage of the Edu positive cells. The improved and restored liver function of patients with fibrosis induced by chronic injury depends on both hepatocyte regeneration/differentiation and mass, thus, FZHY might also work on both processes *in vivo* depending on the pathophysiological conditions of the patients. EMT plays an important role in liver fibrosis initiation and development^17^, hepatic stellate cells as well as portal fibroblasts also play a pivotal role in liver fibrogenesis^2^; however, recent reports indicate that EMT, i.e., formation of these mesenchymal cells from hepatic epithelium, contributes to the process of hepatic fibrosis^17^,^48^,^49^,^50^,^51^,^52^,^53^. There have been several studies reporting on the actions of FZHY in inhibiting liver fibrosis in rodents and in humans^10^,^11^,^12^,^13^, including indications that FZHY may reverse *in vivo* fibrosis through the inhibitions of both the EMT^11^ and the activation of hepatic stellate cells to decrease the formation of myofibroblast^13^. Our study revealed that the markers of mesenchymal cells/EMT, N-cadherin, α-SMA, and Vimentin, and EMT-associated transcription factors, Snail1, and Twist1, were significantly reduced by treatment with FZHY during hepatocyte differentiation and maturation processes, indicating that FZHY appears to inhibit the formation of mesenchymal cells through the reduction of EMT *in vitro*. Thus, our report is consistent with the *in vivo* findings and suggests possible mechanisms of these interactions. Therefore, it is interesting to speculate that FZHY may both promote the proliferation of hepatocytes to restore the liver function in patients with liver disease with moderate fibrosis, as well as enhancing the differentiation of liver stem/progenitor cells to restore liver function in patients with liver disease with more marked fibrosis/cirrhosis. Furthermore, FZHY appears to limit fibrogenesis through the inhibition of forming mesenchymal cells by the reduction of the EMT. Taken together, it appears to represent an excellent therapeutic agent for the treatment of liver fibrosis.

Our results showed improvement of hepatocyte differentiation and maturation through enhancing the canonical (β-catenin dependent) Wnt and ERK signaling pathways, and both pathways employ β-catenin to perform their functions. After the activation of Wnt and ERK, β-catenin is translocated into the nucleus^21^,^22^,^23^. With FZHY treatment, the amount of nuclear β-catenin was increased in the treated cells, and TCF1, a target gene for β-catenin in the nucleus, was also increased. Consequently, TCF1 downstream target genes, cyclin D1 and c-myc, were up-regulated, and both genes facilitate cell proliferation^46^. This increased activation of Wnt and ERK may represent a potential mechanism to explain how our hESC-derived hepatocytes treated with FZHY exhibited enhanced proliferation during the differentiation and maturation stages.

The Notch signaling pathway is necessary for specification of the biliary tree, and Notch pathway ablation results in failure of hepatoblast specification to cholangiocytes^54^,^55^,^56^, based on the finding in patients affected with Alagille syndrome, a polymalformative disease with bile duct paucity^42^,^57^,^58^. Notch receptors (Notch1-4), and Notch ligands (DDLs, Jagged1, 2)^59^, are highly expressed during biliary regeneration of mouse hepatic progenitor cells, indicating that the Notch pathway was activated^60^, and this activation was confirmed by greater expression of both the Notch receptor targets Hes1 and Hey1 in biliary regeneration when compared to hepatocyte regeneration. These results strongly suggest that in hepatocyte regeneration there is restricted activation of the Notch pathway^54^. Furthermore, the Notch pathway effectors Hes5 and HeyL are more highly expressed during biliary regeneration than hepatocyte regeneration^54^, and Notch inhibitor (GS) significantly reduced the expression of Notch effectors Hes1, Hes5, Hey1 and HeyL^54^. The Notch signaling pathway is modified by multiple factors, including Numb, an ubiquitin ligating enzyme, which acts as a negative regulator of the Notch pathway^60^,^61^. In a previous study, Numb promoted the ubiquitination of the Notch1 receptor, which in turn resulted in the degradation of downstream activation of Notch1 target gene Hes1^62^. Down-regulation of endogenous Numb in cultured intestinal cells using RNA interference increased Notch signaling, resulting in the up-regulation of Hes1^63^. Moreover, Numb is a direct tanscriptional target of the Wnt signalling pathway, thus Numb is a key mediator of the coordinated interaction of the Wnt and Notch pathways^24^,^25^,^54^. In our study, FZHY treatment resulted in up-regulation of Numb (Figs 5F and 6F) and the reduction of CK7, Notch receptors (Notch1 and Notch4), Notch ligands (DLL1, DDL3, and Jagged2), and Notch receptor target genes (Hes1 and Hes5), as determined by qPCR and Western bolt (Fig. 4B–E), suggesting that upon ERK and Wnt pathways activation after treatment with FZHY, β-catenin was translocated into the nucleus, where it associated with transcription factor, TCF1, to drive Numb expression. Moreover, up-regulation of Numb negatively regulated the Notch pathway, resulting in inhibition of the biliary differentiation of hESC with FZHY treatment.

**Figure 8:**
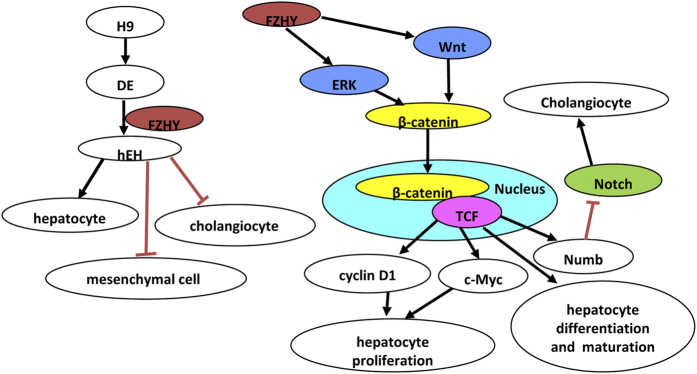
Illustration of the role of FZHY during hepatocyte differentiation. Cartoon illustration of the role of FZHY during hepatocyte differentiation: After the induction of definitive endoderm (DE) from hESC, DE cells were used to differentiate towards hepatocytes (hEH) in conjunction with treatment with FZHY. FZHY enhanced ERK and Wnt signaling pathways, and these two pathways interact at β-catenin. The increase of β-catenin after translocation into nuclei activated TCF1, which up-regulated its downstream genes, consequently enhancing the proliferation, differentiation and maturation of the differentiating cells. TCF1 down-regulated Notch pathway to inhibit the formation of cholangiocytes though up-regulation of Numb. In addition, the treatment with FZHY also inhibited the formation of mesenchymal cells.

In summary, FZHY treatment appears to not only enhance hepatocyte differentiation and maturation, but also promote hESC-derived hepatocyte proliferation. Our results further indicate that such enhancement is likely mediated through activation of the canonical Wnt and ERK pathways; in addition, the inhibition of the Notch pathway suppresses the formation of cholangiocytes by FZHY (Fig. 8). Furthermore, FZHY treatment also inhibited the formation of mesenchymal cells through the reduction of EMT, thus suggesting another possible mechanism by which FZHY may promote hepatocyte differentiation and maturation is through inhibition of the formation of mesenchymal cells (Fig. 8). Our *in vitro* results appear to confirm previous *in vivo* findings in rodents and humans. Therefore, our results not only explain possible mechanism by which FZHY restores the liver faction and inhibits fibrosis in patients, but they also demonstrate that FZHY treatment can enhance our effort to generate mature hepatocytes with proliferative capacity for cell-based therapeutics and for pharmacological and toxicological studies.

## Materials and Methods

### Cell culture

The human embryonic stem cell line, H9, was purchased from WiCell Research Institute (WiCell, Madison, WI), and maintained and expanded on mouse embryonic fibroblasts (MEFs) (GlobalStem, Rockville, MD) as instructed by the provider.

### Differentiation of hESC towards hepatocytes

Hepatocyte differentiation was performed as previously described^15^; briefly, the induction of definitive endoderm (DE) from hESCs was initiated by RPMI medium (Invitrogen, Carlsbad, CA) with 100 ng/ml Activin A (R & D Systems Inc., Minneapolis, MN) without serum for 2 days, and then the medium was changed to RPMI medium with 100 ng/ml Activin A, 0.5 mM sodium butyrate and B27 supplement (Invitrogen) for up to 6 days. DE cells were then split and re-seeded on collagen I-coated 6-well plates (BD Biosciences, San Diego, CA) in hepatocyte differentiation medium (HDM) which contains IMDM media (Invitrogen) supplemented with 20% FBS (Invitrogen), 2 mM L-glutamine (Invitrogen), 0.3 mM 1-thioglycerol (Sigma-Aldrich, St. Louis, MO), 0.5% DMSO (Sigma), 100 nM dexamethasone (Sigma), 0.126 U/ml human insulin (Hospira, Inc), FGF-4 (20 ng/ml), HGF (20 ng/ml), BMP2 (10 ng/ml), and BMP4 (10 ng/ml) (R & D Systems Inc.) for 2 weeks. Then the cells were further differentiated and maturated in hepatocyte culture medium (HCM) which contains hepatocyte basal medium (Lonza, Walkersville, MD) supplemented with SingleQuots kit (Lonza), 0.5% DMSO, 100 nM dexamethasone, 20 ng/ml FGF4, 20 ng/ml HGF, and 50 ng/ml oncostatin M (R & D systems Inc.) until use.

### Treatment of differentiated cells with FZHY

FZHY was prepared and provided by Shanghai Sundise Traditional Chinese Medicine Co., Ltd., China (SFDA approval No: Z20050546, Shanghai, China). The formula of the FZHY per dose is listed (Suppl. Table 1), and ten compounds of FZHY were characterized by chromatographic profiling (Suppl. Fig. 1). Briefly, the following crude herbs were combined to make the FZHY extract powder:, 8.0 g of Danshen, 4.0 g of Dongchongxiacao, 2.0 g of Wuweizi, 2.0 g of Taoren, 2.0 g of Songhuafen, and 6.0 g of Jiaogulan. The quality control and preparation standardization of FZHY is established and enforced strictly by Shanghai Sundise Traditional Chinese Medicine Co., Ltd. In this study, FZHY powder was dissolved in DMSO. FZHY was used to treat the differentiating cells at day 8 and day 20 after the DE cells were re-seeded on collagen I-coated plates for differentiation at the final concentrations of 50 and 100 μg/ml for 6 days respectively; the differentiating control cells were treated with the same concentration of DMSO only.

### Immunohistochemistry analysis

To assess the proliferation of hESC-derived hepatocytes, double-immunostaining was used to detect albumin (ALB) (goat polyclonal antibody, 1:1000, Bethyl Inc., Montgomery, TX) and Ki67 (rabbit polyclonal antibody, 1:500, Abcam, Cambridge, UK). Differentiated cells were fixed with 4% paraformaldehyde and incubated at 4 °C overnight with primary ALB and Ki67 antibodies. The next day, cells were incubated in Cy3-conjugated donkey anti-goat IgG (1:250) or Alexa Fluor® 488-conjugatd goat anti-mouse IgG (1:100) (Jackson Immunoresearch Laboratories, West Grove, PA) at room temperature for 1 hour. Nuclei were stained by 4’,6-Diamidino-2-Phenylindole (DAPI).

### MTT viability assay

The MTT 3-(4,5-dimethyl-2-thiazolyl)-2,5-diphenyl- tetrazolium bromide-based *in vitro* toxicology assay kit was purchased from Sigma-Aldrich. Assay analysis was performed per the manufacture’s instructions. Briefly, MTT was added in an amount equal to 10% of the culture medium volume, and culture media was aspirated 4 hours later. The resulting formazan crystals were dissolved in MTT Solubilization Solution and measured for absorbance at a wavelength of 570 nm.

### Enzyme-Linked Immunosorbent Assay (ELISA) analysis

Every 48 hours after treatment with FZHY, the medium supernatant was collected for determining human ALB secreted into the medium by hESC-derived hepatocytes employing the Human Albumin ELISA Quantitation kit (Bethyl Inc.). The assay was performed according to the manufacturer’s instructions.

### Quantitative RT-PCR analysis

Differentiated cells were harvested at day 14 and day 26 after treatment with FZHY, and total RNA isolation, cDNA generation and quantitative real-time PCR (qPCR) were carried out as previously described^15^. Primers or primers/probes used are listed in Suppl. Table 2.

### Western blot analysis

Differentiated cells treated with or without FZHY were lysed in RIPA buffer with proteinase inhibitor cocktail and phosphatase inhibitor cocktail (Millipore, Billerica, MA) at day 14 and day 26 after differentiaition. 30–50 μg of total proteins or 5–10 μg of nuclear protein was used for Western blot analysis as previously described^19^. Antibodies used are listed in Suppl. Table 3.

### Flow cytometry for Edu cell proliferation analysis

Click-iT® EdU Alexa Fluor® 488 Flow Cytometry Assay Kit (Invitrogen) was employed, and the cell proliferation analysis was performed according to the manufacturer’s instructions. Briefly, the differentiated cells were labelled with an EdU concentration of 40 μM at 37 °C, 5% CO_2_ for 4 h. The cells were harvested and fixed, then 1X saponin-based permeabilization and wash reagent were added to permeate the cells. The reaction cocktail was added and the reaction mixture was incubated for 30 minutes at room temperature, then washed. Finally, the 1X saponin-based permeabilization and wash reagent were added and the cells were analyzed using a flow cytometer.

### PCR Array assays

Differentiated cells were harvested at day 14 after treatment with or without FZHY, and total RNA was isolated and cDNAs were generated using the RT^2^ First Strand Kit (Qiagen, Valencia, CA). The Drug Metabolism Phase I Enzyme RT^2^ Profiler PCR Array (PAHS-068Z), and the Signal Transduction PathwayFinder PCR Array (PAHS-014Z) (all from Sabiosciences, Valencia, CA), were employed using the RT^2^ SYBR Green qPCR Mastermix (Sabiosciences), and data were analyzed as indicated by the manufacturer.

### Statistical analysis

All data were summarized as means ± SEM from at least three independent measurements. An Unpaired Student t-test was used to analyze the data. p < 0.05 was considered statistically significant.

## Additional Information

**How to cite this article**: Chen, J. *et al*. Enhancement of hepatocyte differentiation from human embryonic stem cells by Chinese medicine Fuzhenghuayu. *Sci. Rep*. **6**, 18841; doi: 10.1038/srep18841 (2016).

## Supplementary Material

Supplementary Information
