# Supplementary material for: Enhancement of hepatocyte differentiation from human embryonic stem cells by Chinese medicine Fuzhenghuayu
Source: Sci Rep. 2016 Jan 6;6:18841. doi: 10.1038/srep18841 (PMC4702137; doi:10.1038/srep18841)
Supplement: Supplementary Information [file srep18841-s1.pdf]

Enhancement of hepatocyte differentiation from human embryonic stem cells by Chinese medicine Fuzhenghuayu

Jiamei Chen[1-4] Wei Gao[2,3,5] Ping Zhou[2,3] Xiaocui Ma[2,3] Benjamin Tschudy-Seney[2,3] Chenghai Liu[1], Mark A Zern[2,3] Ping Liu[1], Yuyou Duan[2-4]

[1]Institute of Liver Diseases, Shuguang Hospital, Shanghai University of Traditional Chinese Medicine, Shanghai, China

[2]Department of Internal Medicine, [3]Institute for Regenerative Cures, [4]Department of Dermatology, University of California Davis Medical Center, Sacramento, California, USA

[5]Institute of Biophysics, the Chinese Academy of Science, Beijing, China

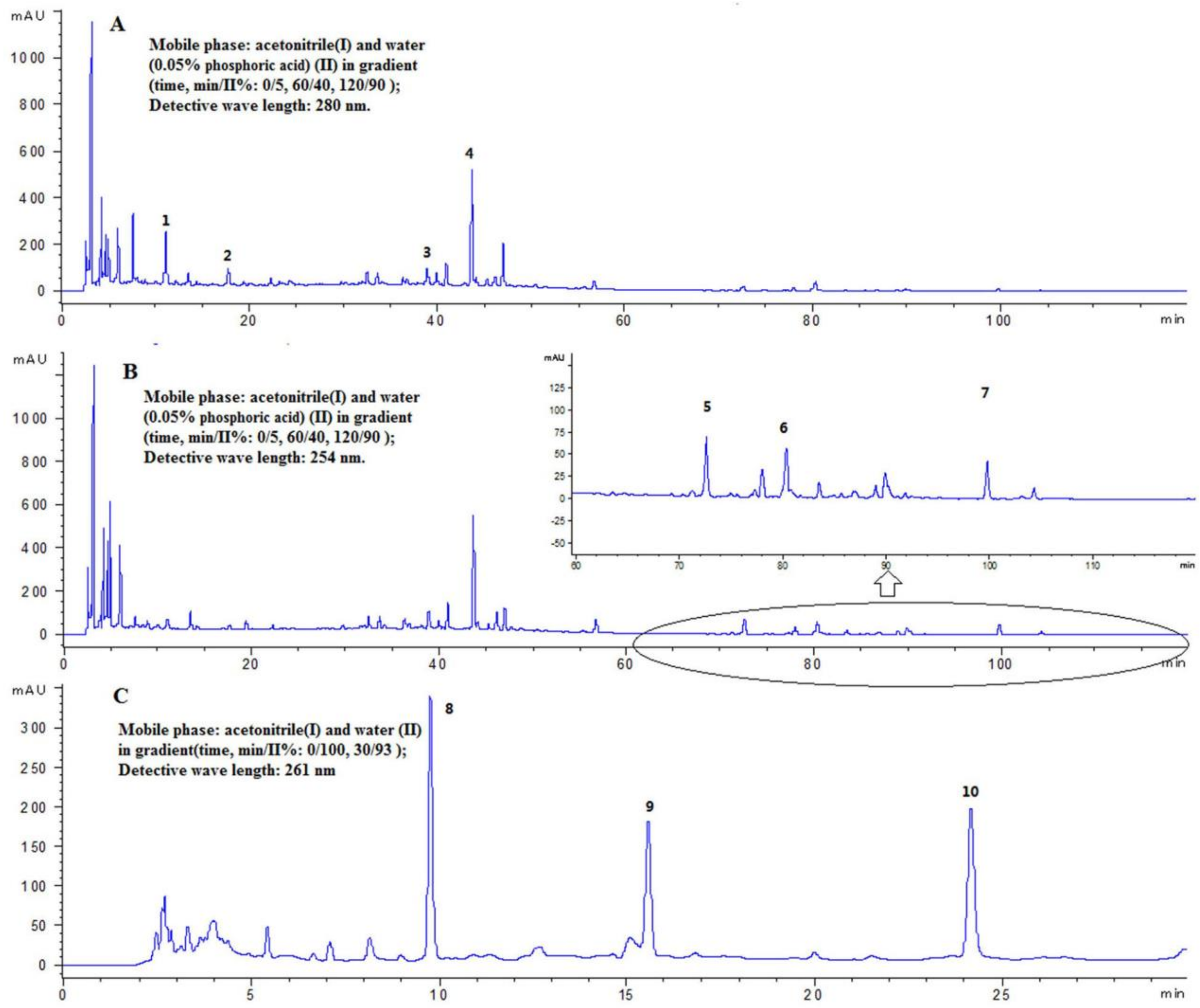

Suppl. Figure 1. The chromatographic profile of FZHY extracts. In a previous study, ten compounds have been identified in FZHY according to the optimized mobile phase employing the chromatography technology, and the chromatographic profiling of FZHY was shown as above. (Stationary phase: CNW Athena C_{18} – WP (4.6mm × 150mm, 3μm), flow rate: 1 mL/min). (Each compound indicated by peak number: 1. danshensu; 2. protocatechuic aldehyde; 3. rosmarinic acid 4. salvianolic acid B; 5. schizandrol A; 6. schizandrol B; 7. schizandrin A; 8. uridine 9. guanosine; 10. adenosine;) [Supplementary Reference 1]

Suppl. Reference 1

1. Yang T, Shen DP, Wang QL et al. Investigation of the absorbed and metabolized components of Danshen from Fuzheng Huayu recipe and study on the anti-hepatic fibrosis effects of these components. J Ethnopharmacol 2013;148:691-700.

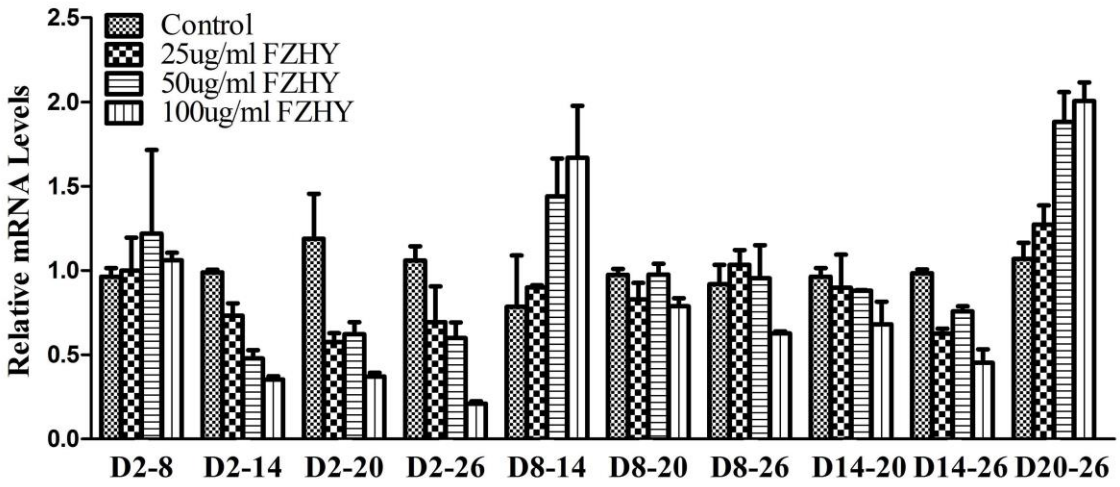

Suppl. Figure 2. Optimization of the treatment with FZHY during hepatocyte differentiation. hESC line, H9 was used in our standard differentiate protocol, and FZHY was added to the culture medium at day 2, 8, 14, 20 of differentiation respectively at different concentrations 25, 50, 100μg/ml, and the cells were collected every 6 days. The relative expression levels of albumin (ALB) was measured by qPCR, and compared to the control groups. Data represent mean ± SEM.

Layout	01	02	03	04	05	06	07	08	09	10	11	12
A<br>100 ug/ml FZHY	AADAC<br>2.04	ADH1A<br>1.40	ADH1B<br>-1.27	ADH1C<br>-1.16	ADH4<br>-1.11	ADH5<br>1.21	ADH6<br>1.56	ADH7<br>1.56	ALDH1A1<br>1.22	ALDH1A2<br>2.74	ALDH1A3<br>-1.27	ALDH1B1<br>-1.26
B<br>100 ug/ml FZHY	ALDH2<br>1.20	ALDH3A1<br>1.61	ALDH3A2<br>1.29	ALDH3B1<br>1.78	ALDH3B2<br>-1.55	ALDH4A1<br>1.37	ALDH5A1<br>1.31	ALDH6A1<br>1.37	ALDH7A1<br>1.09	ALDH8A1<br>1.50	ALDH9A1<br>-1.04	CEL<br>1.38
C<br>100 ug/ml FZHY	CYP11A1<br>-2.52	CYP11B1<br>1.56	CYP11B2<br>1.56	CYP17A1<br>-1.33	CYP19A1<br>1.46	CYP1A1<br>2.97	CYP1A2<br>2.77	CYP1B1<br>1.10	CYP21A2<br>-1.30	CYP24A1<br>-3.74	CYP26A1<br>-1.16	CYP26B1<br>1.34
D<br>100 ug/ml FZHY	CYP26C1<br>1.11	CYP27A1<br>1.26	CYP27B1<br>1.21	CYP2A13<br>-2.16	CYP2B6<br>-1.24	CYP2C18<br>-1.08	CYP2C19<br>2.65	CYP2C8<br>1.72	CYP2C9<br>1.95	CYP2D6<br>1.25	CYP2E1<br>-1.44	CYP2F1<br>2.49
E<br>100 ug/ml FZHY	CYP2R1<br>1.10	CYP2S1<br>1.16	CYP2W1<br>3.11	CYP3A4<br>1.31	CYP3A43<br>1.66	CYP3A5<br>2.31	CYP3A7<br>2.31	CYP4A11<br>1.29	CYP4A22<br>4.38	CYP4B1<br>1.33	CYP4F11<br>3.55	CYP4F12<br>1.94
F<br>100 ug/ml FZHY	CYP4F2<br>-1.90	CYP4F3<br>1.60	CYP4F8<br>1.56	CYP7A1<br>1.85	CYP7B1<br>1.67	CYP8B1<br>1.13	DHRS2<br>-1.13	DPYD<br>1.77	ESD<br>1.38	FMO1<br>1.09	FMO2<br>1.85	FMO3<br>2.26
G<br>100 ug/ml FZHY	FMO4<br>1.34	FMO5<br>1.79	GZMA<br>6.27	GZMB<br>2.37	HSD17B10<br>1.30	MAOA<br>1.19	MAOB<br>1.58	PTGS1<br>2.78	PTGS2<br>1.29	UCHL1<br>-1.18	UCHL3<br>1.38	XDH<br>1.26

Suppl. Figure 3. PCR Array Analysis of Human Drug Metabolism. The 84 genes used in Human Drug Metabolism PCR Array present Phase I Enzymes, and their expression fold change compared to the control group.

The expression changes of 84 genes involved in phase I drug metabolism reactions including oxidation, reduction, hydrolysis, cyclization, and decyclization by PCR Array (PAHS-068Z, Sabiosciences) analysis at day 14 of differentiation of hESCs treated with or without FZHY. The name of each gene and its expression fold change compared to the control group is shown in the same grid. The genes and their representative Drug Metabolism Phase I Enzymes can be found at Sabiosciences website (http://www.sabiosciences.com/rt_pcr_product/HTML/PAHS-068Z.html).

Layout	01	02	03	04	05	06	07	08	09	10	11	12
**A**<br>**100 ug/ml FZHY**	ACSL3<br>**1.26**	ACSL4<br>**1.46**	ACSL5<br>**1.45**	ADM<br>**1.20**	ARNT<br>**1.08**	ATF4<br>**1.26**	AXIN2<br>**-1.11**	BAX<br>**2.94**	BBC3<br>**1.43**	BCL2<br>**-1.01**	BCL2A1<br>**1.47**	BCL2L1<br>**1.26**
**B**<br>**100 ug/ml FZHY**	BIRC3<br>**1.13**	BMP2<br>**1.17**	BMP4<br>**1.02**	BTG2<br>**-1.18**	CA9<br>**-1.48**	CCL5<br>**-1.38**	CCND1<br>**1.13**	CCND2<br>**-1.21**	CDKN1A<br>**-2.02**	CDKN1B<br>**1.34**	CEBPD<br>**1.67**	CPT2<br>**-1.03**
**C**<br>**100 ug/ml FZHY**	CSF1<br>**1.25**	DAB2<br>**1.06**	EGFR<br>**1.50**	EMP1<br>**-2.90**	EPO<br>**1.64**	FABP1<br>**1.39**	FAS<br>**-1.12**	FCER2<br>**1.65**	FOSL1<br>**-1.29**	FTH1<br>**1.14**	GADD45A<br>**-1.29**	**GADD45B**<br>**1.32**
**D**<br>**100 ug/ml FZHY**	GATA3<br>**-1.14**	GCLC<br>**-1.71**	GCLM<br>**1.39**	GSR<br>**-1.39**	HERPUD1<br>**2.07**	HES1<br>**-4.94**	HES5<br>**1.77**	HEY1<br>**1.18**	HEY2<br>**1.71**	HEYL<br>**1.01**	HMOX1<br>**1.35**	ICAM1<br>**-1.03**
**E**<br>**100 ug/ml FZHY**	ID1<br>**1.02**	IFNG<br>**-1.25**	IFRD1<br>**1.14**	IRF1<br>**2.14**	JAG1<br>**-1.04**	LDHA<br>**1.17**	LFNG<br>**2.21**	LRG1<br>**1.51**	MCL1<br>**1.45**	MMP7<br>**-1.15**	MYC<br>**1.63**	NOTCH1<br>**-1.15**
**F**<br>**100 ug/ml FZHY**	NQO1<br>**1.54**	OLR1<br>**1.62**	PCNA<br>**1.02**	PPARD<br>**1.81**	PTCH1<br>**1.22**	RB1<br>**1.27**	SERPINE1<br>**1.06**	SLC27A4<br>**1.31**	SLC2A1<br>**1.12**	SOCS3<br>**-1.40**	SORBS1<br>**1.09**	SQSTM1<br>**1.11**
**G**<br>**100 ug/ml FZHY**	STAT1<br>**1.80**	TNF<br>**-1.33**	TNFSF10<br>**2.21**	TXN<br>**1.28**	TXNRD1<br>**1.51**	VEGFA<br>**1.22**	WISP1<br>**6.68**	WNT1<br>**1.19**	WNT2B<br>**-1.15**	WNT3A<br>**1.11**	WNT5A<br>**1.31**	WNT6<br>**-2.47**

Supple. Figure 4. PCR Array Analysis of Signaling Pathways. The 84 genes used in Signal Transduction PathwayFinder PCR Array present different pathways, and their expression fold change compared to the control group.

The expression changes of 84 genes representing 10 signaling pathways by PCR Array (PAHS-014Z, Sabiosciences) analysis at day 14 of differentiation of hESCs treated with or without FZHY at the concentration of 100 μg/ml. The name of each gene and its expression fold change compared to those without FZHY is shown in the same grid. The genes and their representative pathways can be found at Sabiosciences website (http://www.sabiosciences.com/rt_pcr_product/HTML/PAHS-014Z.html).

Chinese name	Plant sources	Medicinal parts	Amount in preparation (g)
Danshen	*Salvia Miltiorrhizae* Bge. (Labiatae)	radix	8
Chongcao	artifical fermentation cordyceps	mycelia	4
Taoren	*Prunus persica* (L.) Batsch(Rosaceae)	fruit	2
Jiaogulan	*Gynostemma pentaphyllum* (Thunb.) Makino (Cucurbitaceae)	whole herb	6
Songhuafen	*Pinus massoniana* Lamb.(Pinaceae)	pollen	2
Wuweizi	*Schisandrae Chinensis* (Turcz.) Baill.(Magnoliaceae)	fruit	2

Suppl. Table 1. The formula of Fuzhenghuayu per dose

Genes	Information or sequences of primers	Application
Albumin	Hs00609411_m1 (Applied Biosystems)	TaqMan
E-cadherin	Hs01013958_m1 (Applied Biosystems)	TaqMan
CK7	Hs00559840_m1 (Applied Biosystems)	TaqMan
N-cadherin	Hs00169953_m1 (Applied Biosystems)	TaqMan
ASGPR	Hs00155881_m1 (Applied Biosystems)	TaqMan
Desmin	Hs00157258_m1 (Applied Biosystems)	TaqMan
α-SMA	Hs00426835_m1 (Applied Biosystems)	TaqMan
Vimentin	Hs00185584_m1 (Applied Biosystems)	TaqMan
Ki67	Hs01032443_m1 (Applied Biosystems)	Taqman
Snail 1	Hs00195591_m1 (Applied Biosystems)	TaqMan
Twist	Hs01675818_s1 (Applied Biosystems)	TaqMan
CYP1A2	Hs00167927_m1 (Applied Biosystems)	TaqMan
CYP2C9	Hs00426397_m1 (Applied Biosystems)	TaqMan
CYP2C19	Hs00426380_m1 (Applied Biosystems)	TaqMan
UTG1A1	Hs02511055_s1 (Applied Biosystems)	TaqMan
UTG1A3	Hs04194492_g1 (Applied Biosystems)	TaqMan
UTG1A6	Hs01592477_m1 (Applied Biosystems)	TaqMan
UTG1A8	Hs01592482_m1 (Applied Biosystems)	TaqMan
UTG1A10	Hs02516990_s1 (Applied Biosystems)	TaqMan
UTG2B7	Hs00426592_m1 (Applied Biosystems)	TaqMan
Glut2	Hs01096908_m1 (Applied Biosystems)	TaqMan
TAT	Hs00356930_m1 (Applied Biosystems)	TaqMan
GAPDH	Hs99999905_m1 (Applied Biosystems)	TaqMan
Wnt1	F:5'- CTGCAGCGACAACATTGACTT-3'<br>R:5'- GTTGTTGTGAAGGTTCATGAGG-3'	SYBR
cyclin D1	F: 5'-GGTCTGCGAGGAACAGAAGTG-3'<br>R: 5'-TGCAGGCGGCTCTTTTTC-3'	SYBR
c-Myc	F:5'-AGCTCATTTCTGAAGAGGACTTGT-3'<br>R:5'-TTGAGGCAGTTTACATTATGGCTA-3'	SYBR
TCF1	F: 5'-CCCTACTTTTTATCCCTTGTCTCC-3'<br>R: 5'-CTGAGGTGTTACAATAGCTGGATG-3'	SYBR

GAPDH	F: 5'-GAAGATGGTGATGGGATTTC-3'<br>R: 5'-GAAGGTGAAGGTCGGAGTC-3'	SYBR
Notch1	F: GGCCAGAACTGTGAGGAAAATATC<br>R: ACAGTACTGACCTGTCCACTCTGG	SYBR
Notch4	F: CTGTAGTGAGGAGATGACAGCTTG<br>R: GACACACAGTAGTCAGTGCTGGTT	SYBR
DLL1	F: GTACTGTGACGAGTGTATCCGCTA<br>R: GGCTTATGGTGTGTGCAGTAGTTC	SYBR
DLL3	F: ACTCAACAACCTAAGGACGCAG<br>R: GCGTAGATGGAAGGAGCAGATA	SYBR
Jagged 2	F: ACGAGAACTACTACAGCGCCACTT<br>R: TACACACAGCTTCCTTGCACTC	SYBR
Hes1	F: CTGAGCACAGAAAGTCATCAAAGC<br>R: GAGCTATCTTTCTTCAGAGCATCC	SYBR
Hes5	F: TCAGCTACCTGAAGCACAGCAAAG<br>R: TGGAAGTGGTACAGCAGCTTCATC	SYBR
Wnt2	F: ATCTCTGGAGGAAGTACAATGGGG<br>R: TCTCGGTCCCTGATACAGTAGTCT	SYBR
Wnt3a	F: GACTTCCTCAAGGACAAGTACGAC<br>R: TGGGCACCTTGAAGTAGGTGTA	SYBR
Wnt7a	F: AGATCCTGGAGGAGAACATGAAGC<br>R: CGTTGTACTTGTCCTTGAGCACGT	SYBR
Wnt7b	F: CACCTGCTGAAGGAGAAGTACAAC<br>R: CTCAATGTACACCAGGTCTGTCTC	SYBR
Wnt10b	F: GGGCCATCTTCATTGATACCCACA<br>R: GGAGACTTCTCAAAGTAGACCAGC	SYBR

Suppl. Table 2. Information of primers and probes used

Abbreviations: ASGPR: asialoglycoprotein receptor; α-SMA, alpha smooth muscle actin; CYP, cytochrome P450; UGT, UDP-glucuronosyl-S-transferase; Glut2, glucose transporter protein 2; TAT, tyrosine aminotransferase; GAPDH, glyceraldehyde-3-phosphate dehydrogenase; TCF1, T-cell factor 1; DLL1, Delta-like 1; DLL3, Delta-like 3; Hes1, hairy and enhancer of split-1; Hes5, hairy and enhancer of split 5.

Antigen	Type	Provider	Application
Albumin	Goat polyclonal	Bethyl	IHC
Ki67	Rabbit polyclonal	Abcam	IHC
ERK	Rabbit polyclonal	Cell Signaling Technology	WB
p-ERK	Rabbit polyclonal	Cell Signaling Technology	WB
cyclin D1	mouse monoclonal	Cell Signaling Technology	WB
TCF1	Rabbit polyclonal	Cell Signaling Technology	WB
Wnt1	Rabbit polyclonal	GeneTex	WB
Histone H3K27 Trimethylation	Rabbit polyclonal	Epigentek	WB
β-catenin	Rabbit monoclonal	Cell Signaling Technology	WB
Numb	Rabbit monoclonal	Cell Signaling Technology	WB
Hes1	Rabbit monoclonal	Genetex	WB
Jagged2	Rabbit monoclonal	Cell Signaling Technology	WB
N-cadherin	Rabbit monoclonal	Cell Signaling Technology	WB
Vimentin	Rabbit monoclonal	Cell Signaling Technology	WB
c-Myc	Rabbit monoclonal	Santa cruz	WB
GAPDH	mouse monoclonal	Abcam	WB

Suppl. Table 3. List of antibodies used
Abbreviations: ERK, extracellular signal-regulated kinase; p-ERK, phosphorylated ERK; TCF1, T-cell factor 1; Hes1, hairy and enhancer of split-1; GAPDH, glyceraldehyde-3-phosphate dehydrogenase.
